# *In Silico* Drug Screening Analysis against the Overexpression of *PGAM1* Gene in Different Cancer Treatments

**DOI:** 10.1155/2021/5515692

**Published:** 2021-05-31

**Authors:** Muhammad Mazhar Fareed, Mohamed A. El-Esawi, Enas M. El-Ballat, Gaber El-Saber Batiha, Abdur Rauf, Fatma M. El-Demerdash, Fahad A. Alhumaydhi, Suliman A. Alsagaby

**Affiliations:** ^1^Faculty of Life Sciences, Department of Bioinformatics and Biotechnology, Government College University, Faisalabad, Pakistan; ^2^Botany Department, Faculty of Science, Tanta University, Tanta 31527, Egypt; ^3^Department of Pharmacology and Therapeutics, Faculty of Veterinary Medicine, Damanhour University, Damanhour, 22511 AlBeheira, Egypt; ^4^Department of Chemistry, University of Swabi, Anbar, KPK, Pakistan; ^5^Department of Environmental Studies, Institute of Graduate Studies and Research, Alexandria University, Egypt; ^6^Department of Medical Laboratories, College of Applied Medical Sciences, Qassim University, Buraydah, Saudi Arabia; ^7^Department of Medical Laboratory Sciences, College of Applied Medical Sciences, Majmaah University, Majmaah 11932, Saudi Arabia

## Abstract

Phosphoglycerate mutase 1 (PGAM1) is considered as a novel target for multiple types of cancer drugs for the upregulation in tumor, cell prefoliation, and cell migration. During aerobic glycolysis, PGAM1 plays a critical role in cancer cell metabolism by catalyzing the conversion of 3-phosphoglycerate (3PG) to 2-phosphoglycerate (2PG). In this computational-based study, the molecular docking approach was used with the best binding active sites of PGAM1 to screen 5,000 Chinese medicinal phytochemical library. The docking results were three ligands with docking score, RMSD-refine, and residues. Docking scores were -16.57, -15.22, and -15.74. RMSD values were 0.87, 2.40, and 0.98, and binding site residues were Arg 191, Arg 191, Arg 116, Arg 90, Arg 10, and Tyr 92. The best compounds were subjected to ADMETsar, ProTox-2 server, and Molinspiration analysis to evaluate the toxicological and drug likeliness potential of such selected compounds. The UCSF-Chimera tool was used to visualize the results, which shows that the three medicinal compounds named N-Nitrosohexamethyleneimine, Subtrifloralactone-K, and Kanzonol-N in chain-A were successfully binding with the active pockets of PGAM1. The study might facilitate identifying the hit molecules that could be beneficial in the development of antidrugs against various types of cancer treatment. These hit phytochemicals could be beneficial for further investigation of a novel target for cancer.

## 1. Introduction

Cancer has become a serious threat to human life [1]. It was reported that cancer cells always remain in anaerobic glycolysis conditions instead of oxidative phosphorylation [2, 3]. Tumor growth is accomplished through different chemical reactions such as redox and bioenergetic reactions carried out through cancer cells [1]. Metabolic reprogramming is one of the essential parts of cancer cells [2, 3]. The Warburg effect describes the pathway of cancer cells that rely predominantly on the rate of high producing energy by aerobic glycolysis instead of mitochondrial oxidative phosphorylation. The changing of results serves to supply the intermediate of glycolytic actions as building blocks for macromolecules in anabolic biosynthesis, such as lipids, nucleic acids, and proteins, and meet the rapid proliferation requirements of the tumor cells [4]. Thus, targeting key points provide a promising therapeutic method for cancer treatment [5]. The Warburg effect was identified by the increased rate of lactate in cancer cells and glycolysis production in tumor cells as compared to normal cells [4].

Phosphoglycerate mutase 1 (PGAM1) plays a critical role in cancer by the conversion of 3PG to 2PG during glycolysis [6]. PGAM1 is a glycolytic enzyme that dynamically converts 3-phosphoglycerate (3PG) to 2-phosphoglycerate (2PG) and is upregulated to coordinate serine biosynthesis, pentose phosphate pathway (PPP), and glycolysis to regulate tumor and cell proliferation in cancer [7]. PGAM1 is normally expressed in the brain, liver, and kidney tissues [8, 9].

In humans, different types of cancer have been previously identified such as urothelial bladder cancer, breast cancer, renal clear cell carcinoma, hepatocellular carcinoma, lung cancer, colorectal cancer, and liver cancer [10, 11]. Furthermore, PGAM1 has been reported to be associated with proliferation, migration, and apoptosis of tumor cells and its enzymatic activity [12–15]. Prostate cancer (PCa) is the most serious cancer type in males around the world [16]. Recently, PGAM1 as a novel metabolic enzyme against breast cancer was applied to screen for a drug target in chemistry-based functional proteomics [17]. Oral squamous cell carcinoma (OSSC) is characterized by severe high potential progression for both lymphatic metastasis and locoregional invasion [18]. PGAM1 has also been reported to be in association with autoimmune central nervous system disorders. A recent study showed a case in which spermatogenic dysfunction is associated with cell proliferation and apoptosis [19, 20]. PGAM1 plays an important role in anabolic activity to promote the proliferation of cells in cancer and contributes to the development of tumor associated with the glycolysis, and it is used as a therapeutic target potential [21, 22]. The inhibition of PGMA1 results in decreasing the concentration of 2PG and increases the concentration of 3PG in tumor cells. Inhibition assisted by PGMA1-004A leads to the reduction of glycolysis activity to reduce the tumor growth [23]. Hence, PGAM1 is considered to be a targeting role in the cancer therapeutic strategy and inhibited the overexpression of different types of cancer [24].

Bioinformatics has a pivotal role in the identification of cancer genes, mutations, and treatment of disease. The cancer bioinformatics approach provides a platform that assists to treat different types of cancer in multiple ways, according to the specific domains of disease, metabolisms, cell signaling, expression, and proliferation, and to explore the molecular mechanisms of cancer in bioinformatics [25, 26]. The current study was planned to search for the most well-organized PGAM1 inhibitors via *in silico* approach. For this purpose, the library of 5,000 phytochemicals was screened via docking analysis with PGAM1 3D structure at inhibitor sites and successfully identified top three validate compounds. The results suggest opportunities for further optimization of the phytochemicals through *in silico* studies.

## 2. Materials and Methods

### 2.1. Structure Retrieval and Optimization

The 3D structure of PGAM1 protein was retrieved from Protein Data Bank (PDB) using PDB ID: 5Y21. Furthermore, Molecular Operating Environment (MOE) was used for optimizing removing ligand and solvent residue, 3D protonation, and energy minimized of given retrieved structure. This structure was further minimized as a receptor prediction for docking analysis [27].

### 2.2. Ligand Library Preparation

After MOE analysis, about 5,000 phytochemical library was prepared. To find the inhibitor's position of PGAM1, docking has been performed against PGAM1 using software packages of MOE [28].

### 2.3. Refinement of Receptor Protein

The three-dimensional (3D) structure of PGAM1 was taken from PBD using PBD ID: 5y2i [27]. Removal of water molecules and ligand receptor was refined. The minimized given structure was used as a receptor for docking analysis.

### 2.4. Determination of Residues

The attraction of PGAM1 has recognized all residues which participated inefficiently and were selected using the LigX interaction tool of the MOE package [29].

### 2.5. Molecular Docking

The interaction of the selected residues is applied for screening 5,000 Chinese Medicinal phytochemical library extracted through different research literatures, which was developed from the literature search, PubChem, ZINC Database, MPD3 Database, and different drug-ligand databases using MOE software for docking. The following parameters in MOE were set for docking: ligand: MBD file of phytochemicals, placement: triangle matcher; rescoring: London dG: 10; retain: 10: refinement 1: forcefield; rescoring: refinement 2: London dG and retain: 10. The accurate confirmation of ligand is validated to get minimum energy structure. After docking, phytochemicals with top and best confirmation results were determined based on S-score and RMSD-refine values. The MOE's LigX method has been used to evaluate the ligand-receptor interaction 3D plots and is given a cleared view via the docking study of best receptor-residue complexes. The MOE tool used the best three compound interactions with PGAM1 ligand complexes, and receptor complex of PGAM1 was generated through the PyRx Tool [30], and these three top compounds were visualized through binding energy interaction with gene-chain-A via visualizing the UCSF-Chimera tool [31].

### 2.6. Absorption, Distribution, Metabolism, Excretion, ADMET Properties, ProTox-2, and Toxicity Scan

Molinspiration server and ProTox-2 server were used to drug likeliness of proposed PGAM1 inhibitors using an ADMETsar-based drug scan [32, 33]. The selected compounds showed violations by Lipinski's rule of five (Rule-05) and revealed the drug-like properties, i.e., molecular weight. All the selected final compounds were evaluated using the ProTox-2 server based on the toxicity of chemicals of candidates to assess them for their drug-like properties.

## 3. Results and Discussion

**Figure 1:**
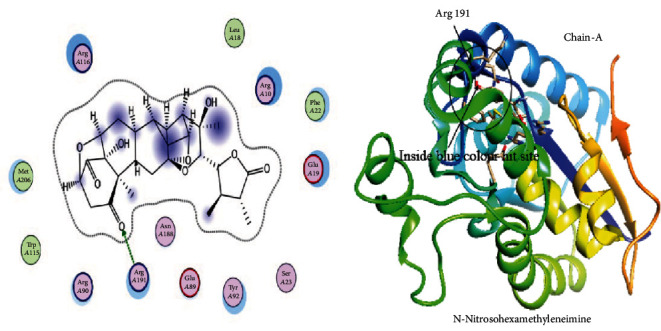
Interaction visualization showing N-Nitrosohexamethyleneimine with binding ligand site of PGAM1 on residue no. Arg 191.

**Figure 2:**
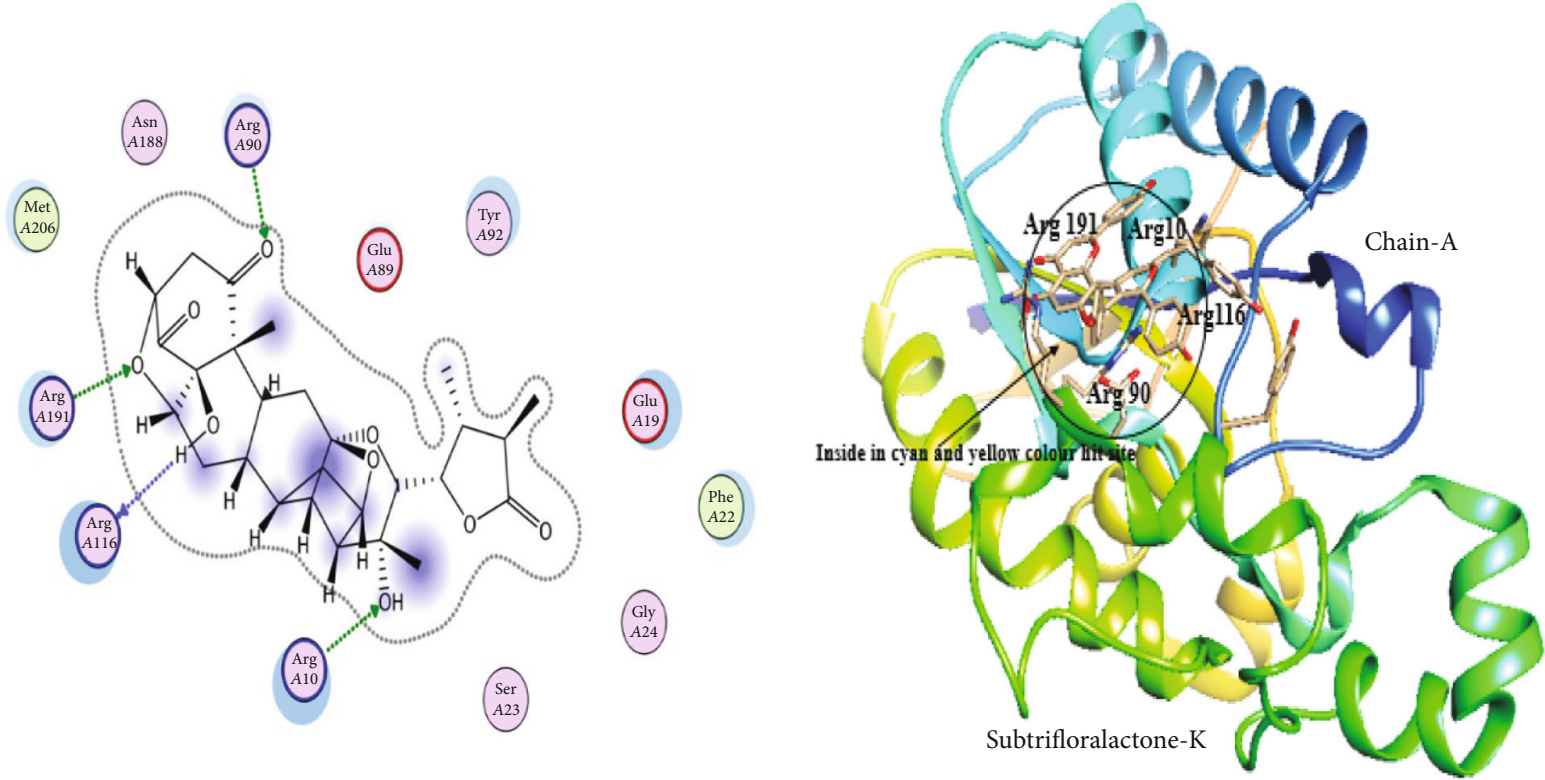
Interaction visualization showing Subtrifloralactone-K with binding ligand sites of PGAM1 on residue no. Arg 191, Arg 116, Arg 90, Arg 10.

**Figure 3:**
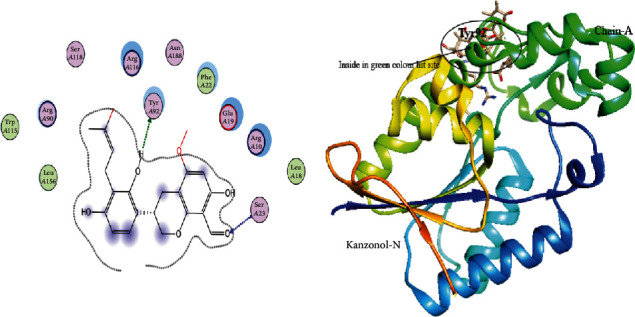
Interaction visualization showing Kanzonol-N with a binding ligand site of PGAM1 on residue no Tyr 92.

The 3D structure of PGAM1 was retrieved from PDB via PDB ID: 5Y2I, which has a resolution of 1.917 Å. This structure was optimized to find the interface and interaction by the MOE site finder tool using default parameters. Docking of PGAM1 with PGMI-004A was performed to dock library of phytochemicals. The interaction residues participating in this reaction of PGAM1 with other ligand proteins are observed by the LigX tool of MOE. The selected library of phytochemical was screened against the PGAM1 protein. In a library of 5,000 Chinese medicinal phytochemicals, three specific docking files were selected. The most hit compound with PGAM1 was selected based on maximum binding sites attached by ligand, lower S-score, and minimum RMSD values with top binding affinity of -7.9, -7.5, and -8.02 in Mol/kcal. These top three phytochemical compounds exhibited their minimum binding energy in the range of -16.7 Kcal/mol to -15.22 Kcal/mol and interaction of RMSD values ranged from 2.40 to 0.87 as shown in Table 1. The top selected ligand complexes were hit against the activity of PGAM1 in cancer cells on the basis of binding energies and ligand activity with target active sites in the structure of PGAM1-chain-A (i.e., N-Nitrosohexamethyleneimine with binding ligand site of PGAM1, Subtrifloralactone-K with binding ligand site of PGAM1, and Kanzonol-N with binding ligand site of PGAM1), and these three top compounds were visualized using visualizing Chimera tool as shown in Figures 1–3.

According to Lipinski's rule [34], the best docking scoring phytochemicals were selected [34]. This followed all phytochemical properties simply done by the Molinspiration server. Selected compound probabilities and ability to pass through the blood barrier, absorption in intestines, metabolism, and distribution at the cellular and subcellular level are shown in Table 2. However, the active binding sites of the PGAM1 have exhibited the binding score and maximum binding affinity and ranked at the top three. After the docking of phytochemical with PGAM1, fined top three compounds tend to exhibit strong binding affinity towards the amino acid residues including Arg 191, Arg 191, Arg 116, Arg 90, Arg 10, and Tyr 92, suggesting the most active site residues with PGAM1. The prediction of genetic toxicity endpoints of candidate compounds (N-Nitrosohexamethyleneimine, Subtrifloralactone-K, and Kanzonol-N) has probability in mutagenicity (0.90, 0,57, and 0.66) and cytotoxicity (0.60, 0.50, and 0.76) (Table 3). The drug-likeness of the three selected compounds was predicted through the Molinspiration server based on Lipinski's rule of five. The selected compounds displayed no violation of Lipinski's rule of five and exhibited drug-like properties (Table 4).

The selected three molecules were then subjected to various toxicity elements. Among three compounds, N-Nitrosohexamethyleneimine obtained no toxicity (LD50 = 336 mg/kg) as class 4: with 100% prediction accuracy; Subtrifloralactone-K obtained no toxicity (LD50 = 90 mg/kg) as class 4: with 69.26%; and Kanzonol-N obtained no toxicity result (LDS50 = 1,250 mg/kg) as class 4: with 68.07% prediction. For the prediction of cytotoxicity with special reference to mutagenicity, all were observed as inactive with a probability score of 0.60, 0.76, 0.50, 0.81, 0.90, 0.57, and 0.66. The prediction results of cytotoxicity and hepatotoxicity revealed that all compounds: N-Nitrosohexamethyleneimine, Subtrifloralactone-K, and Kanzonol-N, were hepatotoxic inactive with probability scores of 0.83, 0.72, 0.76, 0.60, 0.76, and 0.76, respectively, as shown in Table 5.

Computer-based analysis has revolutionized a quick way for drug screening by prominently lowering the difficulty levels as well as by providing all types of requirements of conventional procedure of screening drugs, i.e., drug discovery and simulation approach. The new drug target and potential drugs are being discovered and invented in huge numbers via bioinformatics databases and tools. In this study, we investigate the function of PGAM1 in various types of cancer. PGAM1 is a glycolytic enzyme in nature that catalyzes the conversion of 2-phosphoglycerate and 3-phosphoglycerate [35]. PGAM1 overexpression in both 2PG and 3PG has additional biological functions and is affected in the anabolic condition. In the current study, we examined the cancer cell migration by regulating the mechanism of PGAM1 and tumor growth. We docked an interaction that targeted receptor sites by MOE and inhibited Arg 191, Arg 191, Arg 116, Arg 90, Arg 10, and Tyr 92. These selected compounds can be screened for their drug-likeness and properties using modern computational methods [36]. In the current study, we identified novel-targeted drug-like compounds with desired ADMET characteristics. N-Nitrosohexamethyleneimine, Subtrifloralactone-K, and Kanzonol-N phytochemicals were screened based on their docking score and binding affinity. The significant upregulation of PGAM1 is responsible for cell migration, cell proliferation, tumor growth, and cell division. These inhibitors act as anticancer agents. The agents include small molecule inhibitors, tumor growth receptors, and vaccine-based therapies. The present study identified three inhibitors binding ligand sites with strong potential drugs, efficiently targeting and inhibiting the expression of PGAM1 in cancer.

## 4. Conclusion

In the current study, libraries of phytochemicals including N-Nitrosohexamethyleneimine, Subtrifloralactone-K, and Kanzonol-N with binding site residues are identified as the potential phytochemicals with a strong binding capability with PGAM1 and showed drug-like properties. The findings of this study might be useful for the development and design of potent compounds having better inhibitor-like activities against PGAM1 protein. However, *in vitro* and *in vivo* studies are highly recommended for further investigations.

## Figures and Tables

**Table 1:** Interaction details of top three bioactive phytochemicals in the proposed site of PGAM1 protein.

Sr. no.	Ligands ID	Chemical name	Docking score (S) negative docking value	RMSD value	Residues/receptor
1	33613	N-Nitrosohexamethyleneimine	-16.57	0.87	Arg 191
2	101751351	Subtrifloralactone-K	-15.22	2.40	Arg 191 Arg 116 Arg 90 Arg 10
3	131753028	Kanzonol-N	-15.74	0.98	Tyr 92

**Table 2:** ADMET profiling, absorption, metabolism, and toxicity-related drug-like parameters of candidate compounds.

A. ADMET profiling			
Compounds	N-Nitrosohexamethyleneimine	Subtrifloralactone-K	Kanzonol-N
*A. Absorption*			
Blood-brain barrier	—	+	—
Human intestinal absorption	—	+	+
P-glycoprotein substrate	+	+	—
*B. Metabolism*			
CYP450 1A2 inhibitor	—	—	+
CYP450 2C9 inhibitor	—	—	+
CYP450 2D6 inhibitor	—	—	—
CYP450 2C19 inhibitor	—	—	+
CYP450 3A4 inhibitor	—	—	—
*Distribution*			
Subcellular localization	Lysosomes	Mitochondria	Mitochondria
*Toxicity*			
AMES toxicity	No	No	No

**Table 3:** Prediction of genetic toxicity endpoints of candidate compounds.

Sr. no.	Compound name	Cytotoxicity	Probability	Mutagenicity	Probability
1	N-Nitrosohexamethyleneimine	Inactive	0.60	Inactive	0.90
2	Subtrifloralactone-K	Inactive	0.50	Inactive	0.57
3	Kanzonol-N	Inactive	0.76	Inactive	0.66

**Table 4:** Results of active compounds examined for Lipinski's rule.

Compound	Molecular weight (g/mol)	Number of HBA	Number of HBD	MLogP
Lipinski rule of five	<500	<10	<5	<5
N-Nitrosohexamethyleneimine	128.17	6	3	-2.1
Subtrifloralactone-K	502.55	8	2	0.21
Kanzonol-N	384.42	6	3	4.73

**Table 5:** Prediction of oral acute toxicity, class and accuracy, organ toxicity, and genetic toxicity endpoints of candidate compounds.

Sr. no.	Compound's name	Oral LD50 value (mg/kg)	Predicted toxicity class	Prediction accuracy (%)	Hepatotoxicity	Probability	Cytotoxicity	Probability
1	N-Nitrosohexamethyleneimine	336	4	100	Inactive	0.83	Inactive	0.60
2	Subtrifloralactone-K	90	4	69.26	Inactive	0.72	Inactive	0.76
3	Kanzonol-N	1250	4	68.07	Inactive	0.76	Inactive	0.76

## Data Availability

All the data supporting this study are included in the article.
